# The Forced‐To‐Penetrate Myth Acceptance Scale (FTP‐MAS): A New Attitudinal Tool for Assessing Myths That Surround Female Perpetrated Sexual Violence Against Men

**DOI:** 10.1002/bsl.2706

**Published:** 2024-12-01

**Authors:** Siobhan Weare, Dominic Willmott

**Affiliations:** ^1^ The Law School Lancaster University Lancashire UK; ^2^ Department of Criminology, Sociology and Social Policy Loughborough University Leicestershire UK; ^3^ Faculty of Psychology SWPS University of Social Sciences and Humanities Wroclaw Poland

**Keywords:** confirmatory factor analysis (CFA), female perpetrated sexual assault, forced to penetrate, male victim‐survivors, rape myths

## Abstract

The purpose of this study was to develop and validate a new measurement tool designed to capture endorsement of myths surrounding female perpetrated sexual violence against men, specifically in ‘forced‐to‐penetrate’ cases. Data were collected among a sample of 4152 UK adults aged 18–55+ (52% female). Dimensionality and construct validity of the Forced‐to‐Penetrate Myth Acceptance Scale (FTP‐MAS) was investigated using traditional Confirmatory Factor Analysis (CFA) techniques separately for the complete sample, males only, and females only. CFA results indicated that FTP‐MAS scores are best captured by a three‐factor model (1. Distorted Sex and Gender Roles; 2. Harm Minimisation; 3. Offence Denial) across all samples tested. Excellent composite reliability and differential predictive validity were observed for all three subscales. The validated 22‐item FTP‐MAS constitutes the first measurement tool which allows for the assessment and evaluation of public attitudes towards female perpetrators who force men to penetrate them without consent. As such, this tool enables researchers to better understand the multi‐faceted nature of these myths, assess prevalence in different contexts, and can also be used as an outcome measure in research seeking to evaluate the effectiveness of interventions that aim to debunk endorsement of such myths and stereotypes.

## 1 Introduction

The Crime Survey for England and Wales (CSEW) estimated that 1.2% of men aged 16 years and over had experienced some form of sexual assault (including attempts) in the year ending March 2022 (Office for National Statistics 2023b). Police reported crime data over the same time period highlights that men represented 14.5% of sexual offences victims (Office for National Statistics 2023a). When considering the issue of male sexual victimisation focus tends to be on cases where the perpetrator is male. However, there is a small, yet growing, body of research and data highlighting the issue of female‐on‐male sexual violence and abuse. This research highlights the contexts within which such abuse occurs (e.g., Struckman‐Johnson and Struckman‐Johnson 1994), the impacts on male victim‐survivors (e.g., Weare 2021b), and the different types of sexual abuse experienced by male victim‐survivors (e.g., Stemple and Meyer 2014). Perceptions of male victim‐survivors (e.g., Davies 2013) and myths and stereotypes surrounding this form of sexual violence have also been considered (e.g., Weare 2021a), albeit in significantly less detail than within the context of male‐on‐male and male‐on‐female sexual violence.

This article makes an original and important contribution to knowledge in relation to female‐on‐male sexual violence through the development and validation of a new attitudinal tool examining myths surrounding female perpetrated sexual violence against men, specifically in forced‐to‐penetrate (FTP) cases. The article is structured as follows; the first section defines and explores FTP cases; the second contextualises the development of the attitudinal tool within existing male rape myth acceptance scales; the third details the study aims, methods, analytical procedures, and results; and the final section considers the implications of this research.

## 2 Forced‐To‐Penetrate Cases

Forced‐to‐penetrate (FTP) cases involve a man being forced‐to‐penetrate, with his penis, and without his consent, a woman's vagina, anus, or mouth (Weare 2018). In such cases the perpetrator is a woman, and the victim is a man. The term FTP is used to describe this form of sexual violence because within the UK, the current legal definitions of rape require the male perpetrator to non‐consensually penetrate the victim with his penis (see, e.g. Sexual Offences Act (2003) in England and Wales). As such, it is only men, not women, who can be principal offenders of rape within the UK. Where FTP cases occur, they can be prosecuted under alternative offences. For example, in England and Wales such cases are prosecuted under the offence of ‘causing a person to engage in sexual activity without consent’ (Sexual Offences Act 2003, 4).

The prevalence of FTP cases within the UK cannot currently be accurately determined due to a lack of publicly available data within national crime statistics and within academic research (Weare 2020). However, studies conducted in other jurisdictions have provided insights into the prevalence of this form of sexual offending. Most notably, the 2016/17 *National Intimate Partner and Sexual Violence Survey* in the United States, where data came from interviews with 27,571 adults. The survey found a lifetime prevalence rate for men being made to penetrate of 10.7% (Basile et al. 2022, 3). Of these men, 69.6% per cent reported the perpetrator was female (Basile et al. 2022, 10). Whilst defining ‘made to penetrate’ more broadly than within this research (e.g. also including attempts) (Basile et al. 2022, 2), the findings nevertheless highlight FTP cases as a significant issue with a substantial number of men having experienced this form of sexual violence perpetrated by women.

Smaller scale mixed‐method studies have also highlighted the lived experiences of male victim‐survivors of female‐perpetrated sexual violence. This research has provided insights into the nuances of male sexual victimisation by women (Struckman‐Johnson and Struckman‐Johnson 1994; Krahé, Scheinberger‐Olwig, and Bieneck 2003; Weare and Hulley 2019). Such insights have included the contexts within which such sexual violence most frequently occurs and the relationships between female perpetrators and male victims in FTP cases. Weare and Hulley (2019), for example, found that the 30 men that they interviewed had most frequently been ‘FTP their female partner within an abusive intimate relationship’ (p. 9).

Research has also highlighted the role that myths and stereotypes around men and masculinity, sexual scripts, and sexual violence play in FTP cases (Weiss 2010; Fisher and Pina 2013, Weare 2021a). This is particularly in relation to how male victim‐survivors, and others, understand and process their FTP experiences (Weiss 2010; Weare and Hulley 2019), barriers to disclosing victimisation, and responses to disclosures that may be made by men to family, friends, and professionals (Weare, Hulley, and Craig 2024). These myths and stereotypes include the beliefs that men are sexually insatiable and therefore they welcome all sexual opportunities all of the time, that they cannot be forced or coerced into having sex with a woman against their will, and that consequently all sexual encounters are positive and are never harmful for men (Oswald and Holmgreen 2013; Stemple and Meyer 2014; Weare 2021b). These myths and stereotypes can be directly contrasted with research findings which have highlighted the significant harmful emotional and psychological impacts for male victim‐survivors in FTP cases, including experiencing anxiety, depression, and PTSD, as well as feelings of anger, shame, and distress (Struckman‐Johnson and Struckman‐Johnson 1994; Weare 2021b). These impacts mirror those found in men and boys sexually victimised by varied offender types in both breadth and severity (e.g. Debowska et al. 2024; Sharratt et al. 2023; Widanaralalage et al. 2024), alongside the additional harms that emerge from being victimised by a female offender.

Despite the significant role that myths and stereotypes around men, masculinity, and sexual scripts play in understanding and responding to men's experiences of female‐perpetrated sexual violence, there are no existing measures concerned solely with assessing myths associated with female‐on‐male sexual violence, and therefore none specifically considering FTP cases. It is to this issue that this article now turns.

## 3 Assessing Myths Around Male Sexual Victimisation

Rape myths are ‘prejudicial, stereotyped, or false beliefs about rape, rape victims, and rapists’ (Burt 1980, 217) that have the effect of ‘minimising rape as a serious concern, blaming victims, and defending perpetrators’ (Hogge and Wange 2022, 422). To measure the acceptance of such myths, rape myth acceptance scales have been developed ‘to accurately assess and compare myth acceptance across groups, as well as the predictors and outcomes of rape myth acceptive attitudes’ (Hine, Murphy, and Churchyard 2021, 2). The majority of these scales have focused upon female sexual victimisation, occurring at the hands of male perpetrators for example, the Illinois Rape Myth Acceptance Scale (IRMAS) (Payne el., 1999). However, three measures have been developed to measure rape myth acceptance in relation to male sexual victimisation, albeit they focus principally on male‐on‐male rape and sexual abuse.

The first of the male rape myth scales was created by Struckman‐Johnson and Struckman‐Johnson (1992) who created a twelve‐item scale based on six rape myth statements, with each item being repeated for a male and a female perpetrator. The scale involved short statements reflecting the following three rape myth dimensions; ‘male rape cannot happen’, ‘male victims are to blame for their own rape’ and ‘getting raped doesn't really upset men’ (Struckman‐Johnson and Struckman‐Johnson 1992, 86–87). The scale was administered to university students with them marking their agreement with the statements on a 6‐point Likert scale (1 = *strongly disagree*; 6 = *strongly agree*). Whilst the scale did incorporate questions relating to female perpetrated sexual violence, the narrowness of the range of myths considered is problematic. As noted by Struckman‐Johnson and Struckman‐Johnson (1992) themselves, the study was intended to serve as a ‘preliminary investigation of beliefs about male rape’ (p. 98), with them highlighting a need to assess beliefs in male rape myths with a greater variety of measures. The authors also noted the need to assess the acceptance of male rape myths in community populations, as well as in specialist populations, such as criminal justice, legal, and medical professionals (Struckman‐Johnson and Struckman‐Johnson 1992).

The most well‐known of the existing male rape measures is the Male Rape Myth Scale (MRMS) developed by Melanson (1999). This 22‐item, unidimensional measure includes statements involving both male and female perpetrators. Example items relating to female‐on‐male rape include, ‘most men would not enjoy being raped by a woman’ and ‘most men who are raped by a woman are somewhat to blame for not being more careful’ (Melanson 1999). Similarly to Struckman‐Johnson and Struckman‐Johnson's scale, the measure was tested on university students with agreement being rated on a 6‐point Likert scale (1 = *strongly disagree*; 6 = *strongly disagree*). Only 7 of the 22 scale items explicitly mentions the perpetrator as a woman, meaning that once again the range of myths considered in relation to female on male sexual violence is narrow. Moreover, as noted by Hine et al. in 2021 the items had not been updated since the scale's initial development over 20 years previously, and therefore the age of the scale was ‘a threat to its validity’ (Hogge and Wang 2022, 424), despite its continued contemporary use (see Willmott and Widanaralalage 2024). Perhaps more problematically, the original MRMS was never psychometrically validated, meaning the extent to which the proposed unidimensional conceptualisation accurately represents the observed factorial structure of the tool remains unclear.

Hogge and Wang's 2022 study updated the MRMS by revising the wording of 13 of the original 22 items in an effort to improve clarity and broader construct representativeness (Hogge and Wang 2022). They also added six new items to cover a wider range of male rape myths, one of which explicitly mentions a female perpetrator. The revised scale (the MRMS‐R) is a 16‐item bi‐factor measure, with factor one containing items relating to ‘marginalisation’ of male sexual victimisation, and factor two containing items relating to ‘victim culpability’. Within the final revised scale, only three of the 16 items specifically mentions a female perpetrator—‘it is hard to believe a man who says he has been raped by a woman’, ‘most men who are raped by women are somewhat to blame for not being more careful’ and ‘most men who claim they were raped by women are somewhat to blame for not escaping or fighting off the woman’ (Hogge and Wang 2022, 426). As such, the scale continues to consider a very narrow range of myths in relation to female perpetrators and male victims, with the focus instead being on male‐on‐male sexual violence. The MRMS‐R was tested using undergraduate students who were asked to rate their agreement with each statement on a 6‐point Likert scale (1 = *strongly disagree*; 6 = *strongly agree*). Despite improvements made to the MRMS and the inclusion of construct validity testing (missing during Melanson's scale development), the authors chose not to confirm the factorial structure on the 1999 scale to assess whether Melanson's original conceptualisation of male rape beliefs was indeed valid. Moreover, as noted above, the subsequent validation of the revised scale occurred among a small opportunity sample of undergraduate students, meaning neither the original nor the revised conceptualisation of the MRMS has ever been systematically validated among larger and more representative community samples.

The most recently developed measure is the Male Rape Myth Acceptance Scale (MRMAS) (Hine, Murphy, and Churchyard 2021). This new scale focuses specifically on male‐to‐male sexual violence, and thus includes no statements that explicitly mention a female perpetrator. This was a conscious decision by the authors who note that whilst ‘it can be argued that whilst there are some areas of overlap (e.g. victimisation as a threat to masculinity, misinterpretation of physical arousal as pleasure), many myths about men raped by other men and about men sexually assaulted by women, are distinct and should be measured as such’ (Hine, Murphy, and Churchyard 2021, 4). For completeness, the structure of the scale is nevertheless noted here. The MRMAS is a 38‐item, two factor scale, with factor one containing items relating to blame and factor two containing items relating to minimisation/exoneration. Similarly to the other male rape myth scales, the MRMAS was tested using undergraduate university students who were asked to rate their agreement with each statement on a 7‐point Likert scale (1 = *strongly disagree*; 7 = *strongly agree*).

From the above overview, it is clear that the existing measures that consider myths around male sexual victimisation are limited in their usefulness when considering cases involving a female perpetrator. Where measures have included statements involving female perpetrators alongside those involving male perpetrators, Hine, Murphy, and Churchyard (2021) have argued that this is problematic because ‘they evoke very different affective and cognitive interpretations and reactions’ (p. 4). As such, ‘there is a strong theoretical basis for the separate investigation and measurement of male‐on‐male and female‐on‐male myths’ (Hine, Murphy, and Churchyard 2021, 4). This is further justified when considering the nuances of female perpetrated sexual violence, and the gendered experiences of male victim‐survivors, as well as the gendered myths and stereotypes underpinning understandings of, and responses to, female‐on‐male sexual violence, as noted earlier in the article. Specific myths and stereotypes are associated with FTP cases, for example those relating to distorted sex and gender roles, for example any man that has been forced into sex by a woman will have secretly enjoyed it; those relating to harm minimisation, for example a man being forced to have sex with a woman is not as a bad as a woman being forced to have sex with a man; and those relating to offence denial, for example if a man had an erection he cannot claim that he was forced into sex with a woman without his consent. This study therefore addresses a significant gap in knowledge by developing and validating a new attitudinal tool that solely examines the unique and distinct myths surrounding female perpetrated sexual violence against men, specifically in FTP cases. The development and psychometric validation of a distinct FTP myth acceptance scale will also allow researchers to investigate for the first time whether such myths may influence criminal justice outcomes and in particular, jury decision making when FTP cases go to trial.

## 4 Methods

### 4.1 Scale Development

A deductive and inductive method was used to develop the Forced‐to‐Penetrate Myth Acceptance Scale (FTP‐MAS) and its associated items. Some scale items were developed based on examination of items included in other rape myth measurement tools and research such as the existing MRMS and the MRMAS, the AMMSA Scale by Gerger and colleagues (2007) which focused on female experiences of sexual violence, as well examining the framing of items in Debowska and colleagues' (2019) Victim Responsiveness Assessment. Other items were developed based on empirical research conducted by Weare (2017) and Weare and Hulley (2019), which generated detailed insights from primary data collected on men's FTP experiences from interviews and surveys completed by 180 male FTP victim‐survivors in the UK. Scale items and hypothesised factors were developed collaboratively and iteratively by both authors based upon the aforementioned body of research and theory which pertains to male sexual victimisation. Item language was often adjusted until consensus was held between both authors. To ensure content validity throughout the range of proposed items and associated subscales, two external experts in both sexual violence and psychometric testing were consulted and asked to comment on item wording to ensure that questions were appropriate, interpretable, and relevant to each overarching hypothesised sub‐scales. Following their feedback and subsequent small scale pre‐testing among 10 opportunistically selected colleagues and students revealed some items (*n* = 4) were consistently cumbersome to understand and they were therefore removed prior to data collection among the current sample.

### 4.2 Sample

In order to test the validity of the FTP‐MAS, we sought to obtain a nationally representative sample of UK adults. To achieve this sample, we employed the services of YouGov, a public opinion data collection service with more than three million registered UK users from whom a stratified sample are drawn. To ensure the generalisability of our findings and that a wide spectrum of diverse views from across the UK were sought, YouGov employed stratified sampling techniques with participants proportionally invited to take part based upon age, biological sex, nationality, and social grade. In total, 4315 UK adults completed the survey in full and were included in the final dataset. Self‐reported biological sex data show 2146 participants (51.7%) were female, and 2006 respondents (48.3%) were male. Categorical age data display that 467 participants (11.3%) were aged 18–24, 661 (15.9%) were 25–34, 768 (18.5%) were 35–44, 610 (14.7%) were aged 45–54, with most respondents (1646; 39.6%) aged 55 and above. The vast majority of the sample were English (84.1%), followed by 347 Scottish nationals (8.3%), 204 Welsh (4.9%), and 109 Northern Irish citizens (2.6%). Based on several questions related to occupation and personal income, YouGov calculated social grade data among those who took part. From these data more than half of the sample (2374; 57.2%) can be broadly classified as ‘middle‐class’ (i.e., ABC1), with 1778 respondents (42.8%) alternatively broadly classified as ‘working‐class’ (i.e., C2DE). The demographic profile of study participants was overall representative of the general population of the UK surrounding social grade, age, and biological sex according to the most recent and available census data.

### 4.3 Study Procedures

Based upon the sensitive nature of the survey and questions therein, once identified and invited by YouGov, participants were provided with an overview of the nature of the study and the focus of the survey questions that they would be asked via an online participant information sheet. Here it was made clear to participants that if they chose to take part, they were permitted to omit any question that they were uncomfortable answering or withdraw from the survey entirely at any point, without being required to provide a reason for doing so. Participants were also provided with contact information for free and impartial UK support service organisations in case they felt that they would like to make use of such support, regardless of whether they decided to take part. As anonymity was provided to those who agreed to take part, participants were informed prior to beginning the survey that their data could not be withdrawn after the final questions were completed and their answers submitted. Those who agreed to take part were presented with the study questionnaire which included the complete FTP‐MAS (note: hypothesised subscale items were presented in a random order rather than being listed by items in each factor), alongside other single item questions and attitudinal scales such as the Gender‐Blind Sexism Inventory (more detail below). The questionnaire took between 25 and 45 min to complete, after which respondents were presented with debrief information reiterating the purpose of the study and were again signposted to impartial support service information. Ethical approval for the study was obtained from the research ethics committee at the lead institution and the procedures outlined above adhered with ethics policies as outlined in the British Psychological Society's (2021) code of human research ethics.

### 4.4 Measures

*Forced‐to‐Penetrate Myth Acceptance Scale* (FTP‐MAS). The FTP‐MAS is a statement‐based assessment tool designed to capture the endorsement of myths and stereotypes pertaining to sexual violence, where a male victim is forced into penetrative sexual activity by, and with, a woman, without his consent. The multidimensional scale is composed of 22‐items scored on a six‐point Likert scale (1 = strongly disagree, 2 = disagree, 3 = somewhat disagree, 4 = somewhat agree, 5 = agree, 6 = strongly agree), and consists of three subscales (1. Distorted Sex & Gender Roles, 2. Harm Minimisation, 3. Offence Denial) that encompass the multi‐faceted nature of myths and misconceptions which surround forced‐to‐penetrate male sexual victimisation. The *Distorted Sex and Gender Roles* factor (9 items) is characterised by stereotypes surrounding masculinity and men's perceived enjoyment of sexual activity regardless of consent (e.g., ‘a real man would never complain about having sex with a woman, regardless of how it happened’) and sub‐scale scores range from 9 to 54. The *Harm Minimisation* factor (8 items) is characterised by beliefs which undermine the harm experienced by men as a consequence of their sexual victimisation (e.g., ‘being forced to have sex with a woman is not something that would negatively affect most men’) and sub‐scale scores range from 8 to 48. The *Offence Denial* (5 items) factor is characterised by the endorsement of beliefs that reject the premise that women can force men to have penetrative sex without their consent (e.g., a man's physical strength means that it’s impossible for a woman to make him have sex with her without his consent) and sub‐scale scores range from 5 to 30. Higher sub‐scale scores indicate greater endorsement of each distinct type of forced‐to‐penetrate myths and stereotypes. Scale reliability scores are included in the results section below. For the complete 22‐item scale refer to the Supporting Information S1: appendix section.

*Gender‐Blind Sexism Inventory* (GBSI; Stoll, Lilley, and Pinter 2017) is a 12‐item multi‐dimensional self‐report scale developed to measure attitudes towards gender‐blind sexism with items designed to capture the contemporary ways that sexism operates in an era of post‐gender politics. The tool consists of four sub‐scales designed to reflect the multidimensional nature of contemporary sexist attitudes that include *Abstract Liberalism* (items 1–3) (e.g. item 2 ‘equal opportunity policies benefit women at the expense of men’); *Naturalisation* (items 4–6) (e.g. item 4 ‘women are naturally more emotional than men’); *Cultural Sexism* (items 7–9) (e.g. item 9 ‘it is better to socialise girls to be caregivers and boys to be breadwinners than vice versa’); *Minimisation of Sexism* (items 10–12) (e.g. item 10 ‘sexism is not a major problem in today's society’). Responses were measured on a six‐point Likert scale (1 = ‘Strongly Disagree’ to 6 = ‘Strongly Agree’). Sub‐scale scores ranged from 3 to 18, with higher scores indicating greater acceptance of contemporary sexist beliefs (Cronbach's alpha ranged from 0.83 to 0.92).

### 4.5 Analytical Procedure

Construct validity and dimensionality of the FTP‐MAS was examined using traditional confirmatory factor analysis (CFA) techniques. All models were specified and tested using Mplus version 7.11 (Muthén and Muthén 2015), with Maximum Likelihood Robust (MLR) estimation. Model 1 was a unidimensional solution where all items load onto a single factor. Model 2 was a correlated three‐factor solution where nine items load onto the factor ‘Distorted Sex and Gender Roles’ (items 4, 8, 11, 15, 18, 22, 25, 29, 31), eight items load onto Harm Minimisation (items 5, 9, 12, 16, 19, 23, 26, 30) and five items load onto Offence Denial (items 3, 10, 13, 17, 27). The overall fit of each model and the relative fit between models was assessed using a range of goodness‐of‐fit statistics. This includes the Comparative Fit Index (CFI; Kline 2015) and the Tucker Lewis Index (TLI; Tucker and Lewis 1973), where values above 0.90 indicate acceptable model fit, and values above 0.95 indicate good model fit (Bentler 1990; Hu and Bentler 1999). In addition, the Standardised Root Mean Square Residual (SRMR) and Root Mean Square Error of Approximation (RMSEA; Steiger 1990), with 90% confidence intervals (CI), are presented. Values below 0.08 indicate acceptable model fit and values below 0.05 indicate good model fit (Bentler 1990; Hu and Bentler 1999). Akaike Information Criterion (AIC; Akaike 1974) was used to compare relative fit between competing models, with the smallest value indicating the best fitting model. Finally, due to the potential for Cronbach's alpha to over‐ or under‐estimate internal consistency, composite reliability was computed in the present analysis using the formula provided by Raykov (1998). Values above 0.60 are considered acceptable (Diamantopoulos and Siguaw 2000). Previously authors (e.g. Boduszek and Debowska 2016; Sherretts and Willmott 2016) have recommended that multi‐factorial measurement scales where sub‐scales may be highly correlated (0.50 and above) should examine the differential predictive validity of distinct sub‐scales to verify that the factors correlate differently with external variables. Based on the full sample, we applied structural equation modelling (SEM) to assess the utility of a three‐factor solution in predicting factor scores on the Abstract Liberalism, Naturalisation, Cultural Sexism and Minimisation of Sexism subscales of the Gender‐Blind Sexism Inventory (GBSI).

## 5 Results

Descriptive statistics and gender differences for the three FTP‐MAS subscales (Distorted Sex and Gender Roles, Harm Minimisation, Offence Denial), and the four GBSI subscales (Abstract Liberalism, Naturalisation, Cultural Sexism, Minimisation of Sexism) are presented in Table 1. Results display that male participants exhibited significantly higher scores that their female counterparts on all FTP‐MAS and GBSI subscales. The degree of difference was small for all subscales except minimisation of sexism, where for men a medium difference was observed.

**TABLE 1:** Descriptive statistics and gender differences in FTP‐MAS and GBSI subscales.

Variables	Full sample *M* (SD)	Male *M* (SD)	Female *M* (SD)	*t* (*d*)
Distorted sex and gender roles	15.93 (7.32)	17.67 (7.87)	14.72 (6.54)	13.00*** (0.42)
Harm minimisation	14.84 (7.04)	16.15 (7.59)	13.99 (6.39)	9.84*** (0.31)
Offence denial	9.96 (4.85)	10.44 (5.03)	9.77 (4.70)	4.43*** (0.14)
Abstract liberalism	7.73 (3.52)	8.60 (3.71)	6.92 (3.13)	15.71*** (0.49)
Naturalisation	9.93 (3.22)	10.32 (3.24)	9.56 (3.16)	7.67*** (0.24)
Cultural sexism	8.83 (3.34)	9.51 (3.36)	8.20 (3.19)	12.93*** (0.40)
Minimisation of sexism	9.40 (3.47)	10.34 (3.52)	8.52 (3.18)	17.40*** (0.54)

*Note:* ***, statistically significant at *p* < 0.001; *d*, Cohen's *d* (0.2–0.49 = small differences; 0.5–0.79 = medium differences).

Fit indices for the two proposed models of the FTP‐MAS, tested separately for males, females, and the full sample, are presented in Table 2. In comparison to the unidimensional model, the three‐factor model achieved higher CFI and TLI values, and lower RMSEA and RMSR values across all samples tested, indicating that the three‐factor model provided a better fit for the data. The AIC values were also lower for the three‐factor model than the unidimensional model among the full sample, males only and females only, further confirming that the three‐factor model was the better solution. The three‐factor model was acceptable across all samples according to the RMSEA and SRMR statistics. The CFI statistic indicated that the three‐factor model provided acceptable fit for the data from males, but narrowly missed the threshold for acceptable fit among the full sample and females. The TLI statistic narrowly missed the criteria for acceptable model fit among the full sample and males and was somewhat lower among females. Overall, consideration of all fit indices in combination indicated that the three‐factor solution provided the best fit to the data across males, females, and the complete sample.

**TABLE 2:** Fit Indices for two alternative models of the FTP‐MAS.

	*χ* ^*2*^ (*df*)	CFI	TLI	RMSEA (95% CI)	SRMR	AIC
Unidimensional model						
Full sample	38,529.427 (231)	0.875	0.862	0.074 (0.073/.076)	0.042	176,969.043
Male	19,495.203 (231)	0.896	0.885	0.071 (0.068/.074)	0.035	86,481.081
Female	19,817.639 (231)	0.855	0.839	0.078 (0.076/.081)	0.050	88,109.650
Three‐factor model						
Full sample	38,529.427 (231)	0.887	0.873	0.071 (0.069/.073)	0.040	175,742.769
Male	19,495.203 (231)	0.901	0.889	0.069 (0.067/.072)	0.034	86,195.082
Female	19,817.639 (231)	0.872	0.857	0.074 (0.071/.076)	0.047	87,180.402

The adequacy of the three‐factor model can also be determined based on examination of the factor's loadings (parameter estimates). As shown in Table 3, all factor loadings were statistically significant and exceeded the 0.4 threshold among all samples. Discrepancies in the factor loadings between males and females were small (not exceeding 0.05), suggesting that the items indexed the intended latent constructs similarly well between males and females.

**TABLE 3:** Standardised factor loadings for the three FTP‐MAS factors.

FTP‐MAS items	Full sample	Male	Female
Distorted sex and gender roles			
Q1: Any man that has been forced into sex by a woman will have secretly enjoyed it	0.82	0.82	0.82
Q2: If a man doesn't set clear sexual boundaries then he shouldn't complain that he has been forced into sex by a woman	0.73	0.71	0.75
Q3: It's a man's role to have sex with his wife when she asks for it, whether he wants to or not	0.78	0.76	0.79
Q4: A real man would never complain about having sex with a woman, regardless of how it happened	0.88	0.87	0.87
Q5: Any man that allows a woman to sexually assault him is not a real man	0.83	0.80	0.85
Q6: If a man is forced into sex by a woman he must have wanted it	0.89	0.90	0.89
Q7: If a woman demands sex from her boyfriend or husband, he should be willing to provide it whether he wants to or not	0.79	0.77	0.80
Q8: If a woman demands sex from a man who she is not in an intimate relationship with, he should be willing to provide it whether he wants to or not	0.76	0.76	0.75
Q9: A woman has a right to make her boyfriend have sex with her if they're in a sexual relationship	0.79	0.79	0.78
Harm minimisation			
Q10: Being forced to have sex with a woman is not something that would negatively affect most men	0.74	0.73	0.73
Q11: A man forced to have sex with a woman should be happy, not complain	0.85	0.86	0.84
Q12: A man being forced to have sex with a woman is not as bad as a woman being forced to have sex with a man	0.72	0.73	0.70
Q13: If a man ejaculates whilst a woman is forcing him to have sex, then he must have enjoyed it	0.84	0.86	0.83
Q14: A real man wouldn't be negatively affected if a woman made him have sex against his will	0.86	0.87	0.83
Q15: A real man would enjoy it if a woman forced him to have sex	0.89	0.90	0.88
Q16: If a man maintains an erection whilst being sexually assaulted by a woman, then he must have enjoyed it	0.88	0.89	0.87
Q17: Men who complain that they have been forced into sex by a woman should just be happy that they got to have sex	0.85	0.87	0.83
Offence denial			
Q18: There is no such thing as a man being forced to have sex with a woman against his will	0.79	0.78	0.79
Q19: If a man had an erection he cannot claim that he was forced into sex by a woman without his consent	0.89	0.87	0.90
Q20: A man's physical strength means that it’s impossible for a woman to make him have sex with her without his consent	0.84	0.86	0.84
Q21: Women are not able to make men have sex with them without their consent	0.76	0.77	0.75
Q22: If a man has an erection this means that he is willing to have sex with a woman	0.89	0.89	0.88

*Note:* All factor loadings are statistically significant at ***p < 0.001.

Tests of factorial invariance were conducted between males and females using the three‐factor solution as the baseline model. Following the procedure of Bollen (1989), a hierarchy of increasingly restrictive models were specified and tested. To determine whether the FTP‐MAS was invariant, the model was first fitted without any invariance constraints (configural model). This test of invariance of form, or that this three‐factor model held in both samples, achieved reasonable support (*χ* ^*2*^ (434) = 5053.060, *p* < 0.001; CFI = 0.884; TLI = 0.871; RMSEA = 0.072 [90% CI 0.71/.74]; SRMR = 0.052), as did the tests of equal factor loadings (*χ* ^*2*^ (434) = 5053.060, *p* < 0.001; CFI = 0.881; TLI = 0.873; RMSEA = 0.072 [90% CI 0.70/.73]; SRMR = 0.055), and equal factor variances/covariances (*χ* ^*2*^ (459) = 5594.323, *p* < 0.001; CFI = 0.868; TLI = 0.867; RMSEA = 0.073 [90% CI 0.72/.75]; SRMR = 0.070).

Correlations between the three FTP‐MAS factors were high and statistically significant (*p* < 0.001) among the full sample (*r* = 0.926 to 0.997), males only (*r* = 0.951 to 0.996) and females only (*r* = 0.908 to 0.997). This raises questions about the unique predictive validity of each of the factors, namely their ability to explain variance in outcome variables over and above a single unidimensional factor. As suggested by Boduszek and Debowska (2016) and Willmott and colleagues (2018), when the final model is multi‐dimensional and factors are highly correlated (0.50 and above), a differential predictive validity test should be conducted to verify that the factors correlate differently with external variables. Based on the full sample, structural equation modelling (SEM) was applied to assess the utility of a three‐factor solution in predicting factor scores on the Abstract Liberalism, Naturalisation, Cultural Sexism and Minimisation of Sexism subscales of the Gender‐Blind Sexism Inventory (GBSI). The model achieved reasonable fit (χ2 (506) = 7896.586; CFI = 0.892; TLI = 0.881; RMSEA = 0.059 [90% CI 0.058–0.061]; SRMR = 0.045) and explained a moderate percentage of the variance in Abstract Liberalism (*R*2 = 0.20), Naturalisation (*R*2 = 0.22), Cultural Sexism (*R*2 = 0.43) and Minimisation of Sexism (*R*2 = 0.14). Harm Minimisation demonstrated a positive association with Abstract Liberalism (*β* = 2.091; *p* = 0.033), Naturalisation (*β* = 1.81; *p* = 0.040) and Cultural Sexism (*β* = 1.78; *p* = 0.040). Meanwhile, Offence Denial demonstrated a negative association with Abstract Liberalism (*β* = ‐ 0.79; *p* = 0.016) and Cultural Sexism (*β* = ‐ 0.63; *p* = 0.029). Distorted Sex and Gender Roles was not significantly correlated with any of the GBSI factors. Therefore, it can be seen that FTP‐MAS factors do correlate differently with external variables, confirming the multidimensional nature of the scale, and distinct nature of the three sub‐scales.

To assess the internal reliability of the FTP‐MAS, tests of composite reliability were performed. As shown in Table 4, composite reliability estimates were excellent for all subscales among the full sample (0.92–0.94), males only (0.92–0.95) and females only (0.92–0.95).

**TABLE 4:** Composite reliability for FTP‐MAS factors for full, male and female samples.

Variables	Full sample	Male	Female
Distorted sex and gender roles	0.94	0.94	0.95
Harm minimisation	0.94	0.95	0.94
Offence denial	0.92	0.92	0.92

## 6 Discussion

International data has highlighted FTP cases as being a significant issue, with a substantial number of men having experienced this form of sexual violence perpetrated by women (Basile et al. 2022). Research has also highlighted the gendered myths, stereotypes, and sex role scripts which underpin understandings of, and responses to, FTP cases (Weiss 2010; Fisher and Pina 2013; Weare 2021a). Existing rape myth acceptance measures have typically focused upon female sexual victimisation, something that is unsurprising when considered within the context of women being disproportionately affected by sexual violence overwhelmingly perpetrated by men (Willmott et al. 2021). The few measures that explore male sexual victimisation largely do so in the context of cases where the perpetrators are also male. The lack of existing measures which sufficiently capture the myths associated with female‐on‐male sexual violence, specifically in FTP cases, necessitated the creation of a novel FTP‐MAS. Drawing upon Weare's findings from qualitative research with male FTP victim‐survivors, and an examination of existing measures, the main objective of the current study was to develop a valid and reliable attitudinal tool that captures the unique and wide‐ranging myths surrounding men's experiences of being FTP a woman without their consent. In doing so we evaluated the dimensionality and construct validity of the proposed FTP‐MAS using CFA among a large nationally representative sample of UK citizens, recruited using quota sampling techniques.

It has been suggested that to fully explore the factorial structure of any proposed new measure, theoretically driven alternate solutions should be tested (Boduszek and Debowska 2016; Boduszek et al. 2022). In the current study two alterative models of the FTP‐MAS scale were identified and tested separately among the complete sample, male only sample, and female only sample. This included a one‐factor unidimensional model (i.e., where all FTP myths load onto one single general myth acceptance factor), and a three‐factor multidimensional model (i.e., where FTP myths are grouped around three distinct sub‐factors of FTP myth acceptance). Analysis indicates that the best solution for the 22‐item FTP‐MAS (as indicated by all model fit statistics above) was the three‐factor model (Distorted Sex and Gender Roles, Harm Minimisation, Offence Denial), among all three samples tested.

Next, given some possible conceptual overlap between the three FTP‐MAS factors, the need to establish differential predictive validity between sub‐scales on a multidimensional scale is considered advantageous (Carmines and Zeller 1979). Essentially, ensuring FTP sub‐scales measure separate theoretical, as opposed to statistical, factors by establishing differential associations with gender blind sexism inventory factors allows conceptual distinctiveness to be reliably ascertained (Boduszek and Debowska 2016). With the different FTP sub‐scales exhibiting varied relationships (i.e., positive, negative, and no association) with the GBSI sub‐scales, FTP‐MAS factors clearly therefore do correlate differently with external variables, confirming the multidimensional nature of the scale. Finally, to ensure that the proposed three factorial structure of the scale was equally as useful as an attitudinal assessment tool, and valid among both males and female respondents, factorial invariance were examined between male and female samples separately. Here results indicate that the three‐factorial solution of the FTP‐MAS held consistent, regardless of the sex of the respondents, with sub‐scales items consistently loading onto the same three factors. Taken together, the combination of analysis conducted supports the hypothesised three factor structure of the FTP‐MAS scale, with substantial support for the measure's utility among both male and female samples as a valid assessment of myths and beliefs pertaining to scenarios where men are FTP a woman without their consent.

### 6.1 Limitations and Strengths

As with all research, this study is not free from limitations. The most pertinent of these is that the FTP‐MAS is a self‐report measure, and as such it is associated with possible response bias. Other studies where new attitudinal scales have been developed also encounter similar challenges. However, unlike other studies where new attitudinal scales have been developed, which typically rely on relatively small and opportunistic samples in their validation, often comprised exclusively of homogeneous groups such as university students (e.g., Hine, Murphy, and Churchyard 2021), the sample utilised here is both large and representative. Indeed, a nationally representative sample of over 4000 UK adult participants was utilised in this study, helping to ensure the scales' utility as an accurate assessment of FTP myth acceptance. Moreover, it is noteworthy that the scale items and sub‐scales were theoretically driven and developed based on findings from qualitative data gathered directly via interviews and questionnaires with male FTP victim‐survivors. This is particularly important within the context of this form of sexual violence, where there is limited understanding about men's experiences, especially when compared to the volume of research relating to women's experiences of male perpetrated sexual violence. Next, the FTP‐MAS items relate exclusively to heteronormative scenarios, with the scale validated separately based upon biological sex data. This was reflective of the nature of FTP cases. However, future research should seek to examine differences in FTP‐MAS attitudes among varied gender identity populations as well as those with different sexual orientations in order to consider differences that may exist between such distinct groups. This is especially relevant when the impact of sexual violence myths are considered in legal decision‐making where gender‐differences and biases are often found to exist (e.g. Conroy et al. 2023; Lewandowicz‐Machnikowska et al. 2024).

### 6.2 Implications

The development of the FTP‐MAS has important implications both for research and for practice. First, the scale is novel, and indeed is currently the only means by which myths and attitudes associated with this specific form of sexual violence can be assessed. The importance of having such a measure is reflected in the seminal nature of other rape myth acceptance scales, particularly those which have assessed myths associated with male‐on‐female sexual violence, that have been used in a myriad of ways across a range of diverse groups and populations. Indeed, the FTP‐MAS can be utilised similarly, as a standardised attitudinal assessment tool to assess myths and attitudes associated with this form of sexual violence amongst diverse groups and populations. This could range from specialist populations, such as criminal justice participants (e.g., defendants, complainants, and criminal justice professionals), through to subgroups of the public, for example, young people. As Hine, Murphy, and Churchyard (2021) note, ‘examining the beliefs, judgements, and actions of those in specialist populations … provides an important and highly impactful route to improving the experiences of male victims’ (p. 16). For example, the scale can be used to examine whether criminal justice stakeholder decision making in FTP cases is impacted by pre‐existing beliefs held in relation to this form of female‐on‐male sexual violence. In fact, whilst numerous studies have explored such a relationship in criminal cases involving female rape complainants (Chalmers, Leverick, and Munro 2022; Lilley, Willmott, and Mojtahedi 2023; Ostermann and Watson 2024; Stevens, Mojtahedi, and Austin 2024), no studies to date have directly explored the link between FTP myths, legal decision‐making, and trial outcomes in cases involving male victim‐survivors.

Similarly, the scale can be used within the context of research related to jury decision making in FTP cases. Within England and Wales, it is not possible to conduct research with genuine trial jurors due to provisions within the Juries Act 1974 (as amended by the Criminal Justice and Courts Act 2015), which prevent researchers from asking jurors post‐trial to disclose specific details surrounding how their decisions were reached or discussions that took place during group deliberations (Juries Act 1974, s. 20D). Therefore, mock jury trials represent the best method possible to gain valuable insights into jury deliberations within England and Wales. Within other jurisdictions, for example North America, however, it is possible to conduct research with juror's post‐trial. Within both contexts—mock and real jury trial research—the FTP‐MAS scale can be utilised to examine the beliefs held by jurors at various points within the trial process. For example, pre‐trial to ascertain the impacts that FTP myths may have upon juror decision making during a trial, and post‐trial to understand whether myths and stereotypes have been impacted by trial and deliberative participation. Understanding jury decision making and the factors that can impact decisions is particularly important within the context of serious sexual offences cases because of the low conviction rates in such cases (Willmott and Hudspith 2024). As such, the development and use of the FTP‐MAS provides an opportunity to further develop knowledge and understanding in relation to jury decision making in cases involving male complainants.

The FTP‐MAS also has the potential to be used within intervention and prevention programmes designed to specifically address stereotypes associated with FTP cases, by allowing the efficacy of such programmes to be evaluated in a standardised way. For example, the scale could be administered to programme participants immediately pre‐ and post—their involvement or be utilised in longer term evaluations of the potential enduring efficacy of such programmes.

Finally, it is hoped that the development of the FTP‐MAS ‘provides a previously unavailable avenue to more rigorous, empirical assessment’ (Hine, Murphy, and Churchyard 2021, 16) in relation to female perpetrated sexual violence against men. As a currently under researched area, further exploration of FTP cases, as well as other forms of female perpetrated sexual violence against men, are important to improve knowledge and understanding in this area. In turn it is hoped that this will improve the experiences of male victim‐survivors, and the responses of the criminal justice system in such cases. Similarly, it is hoped that the FTP‐MAS will allow further understanding of societal perceptions of this form of sexual violence to be captured, as well as informing the development of educational campaigns to raise awareness and combat societal misperceptions around FTP cases. The ultimate aim being to improve societal responses to, and understandings of, men's FTP experiences. The importance of this is highlighted within research which has found that many male victim‐survivors feel ‘isolated and unable to discuss their FTP experiences because of the silence that exists around this form of sexual violence … [something] which is further reinforced by powerful and pervasive gender and sex stereotypes around men and masculinity’ (Weare and Hulley 2019, 20).

## 7 Conclusion

In this study we conceptualised, developed, and validated a new attitudinal measurement scale to capture myths towards FTP cases, where men are victims of female perpetrated sexual violence. Developing a separate scale to specifically capture the myths associated with FTP cases recognises the gendered nuances underpinning such cases where the perpetrator is a female, as well as the gendered experiences of male victim‐survivors of this form of female‐perpetrated sexual violence. As the first attitudinal measure of its kind, it is now possible to utilise the FTP‐MAS to assess myths and stereotypes held among varied populations in relation to an often overlooked and under‐researched form of sexual violence. Researchers and practitioners now have a reliable standardised measure by which myths in FTP cases can be better understood, as well as a way in which to evaluate interventions designed to address such myths and stereotypes.

## Conflicts of Interest

The authors declare no conflicts of interest.

## Supporting information

Supporting Information S1
