# Supplementary material for: The Forced‐To‐Penetrate Myth Acceptance Scale (FTP‐MAS): A New Attitudinal Tool for Assessing Myths That Surround Female Perpetrated Sexual Violence Against Men
Source: Behav Sci Law. 2024 Dec 1;43(1):158–68. doi: 10.1002/bsl.2706 (PMC11771554; doi:10.1002/bsl.2706)
Supplement: Supplementary file 1 — Supporting Information S1 [file BSL-43-158-s001.pdf]

Appendix

Weare and Willmott - Forced-to-Penetrate Myth Acceptance Scale [FTP-MAS]

**Distorted Sex & Gender Roles**
1. Any man that has been forced into sex by a woman will have secretly enjoyed it
2. If a man doesn't set clear sexual boundaries then he shouldn't complain that he has been forced into sex by a woman
3. It's a man's role to have sex with his wife when she asks for it, whether he wants to or not
4. A real man would never complain about having sex with a woman, regardless of how it happened
5. Any man that allows a woman to sexually assault him is not a real man
6. If a man is forced into sex by a woman he must have wanted it
7. If a woman demands sex from her boyfriend or husband, he should be willing to provide it whether he wants to or not
8. If a woman demands sex from a man who she is not in an intimate relationship with, he should be willing to provide it whether he wants to or not
9. A woman has a right to make her boyfriend have sex with her if they're in a sexual relationship
**Harm Minimisation**
10. Being forced to have sex with a woman is not something that would negatively affect most men
11. A man forced to have sex with a woman should be happy, not complain
12. A man being forced to have sex with a woman is not as bad as a woman being forced to have sex with a man
13. If a man ejaculates whilst a woman is forcing him to have sex, then he must have enjoyed it
14. A real man wouldn't be negatively affected if a woman made him have sex against his will
15. A real man would enjoy it if a woman forced him to have sex
16. If a man maintains an erection whilst being sexually assaulted by a woman, then he must have enjoyed it
17. Men who complain that they have been forced into sex by a woman should just be happy that they got to have sex
**Offence Denial**
18. There is no such thing as a man being forced to have sex with a woman against his will
19. If a man had an erection he cannot claim that he was forced into sex by a woman without his consent
20. A man's physical strength means that it's impossible for a woman to make him have sex with her without his consent
21. Women are not able to make men have sex with them without their consent
22. If a man has an erection this means that he is willing to have sex with a woman

Note: *the scale is designed to be measured on a six-point Likert scale. Refer to the measures section of Weare and Willmott's article for more information.*
